# Improving Australian National Bowel Cancer Screening Program outcomes through increased participation and cost-effective investment

**DOI:** 10.1371/journal.pone.0227899

**Published:** 2020-02-03

**Authors:** Joachim Worthington, Jie-Bin Lew, Eleonora Feletto, Carol A. Holden, Daniel L. Worthley, Caroline Miller, Karen Canfell

**Affiliations:** 1 Cancer Research Division, Cancer Council NSW, Woolloomooloo, Australia; 2 Prince of Wales Clinical School, University of New South Wales, Sydney, NSW, Australia; 3 South Australian Health & Medical Research Institute, North Terrace, South Australia, Australia; 4 University of Adelaide, Adelaide, South Australia, Australia; 5 Sydney School of Public Health, University of Sydney, Sydney, NSW, Australia; University of Central Florida, UNITED STATES

## Abstract

**Background:**

The Australian National Bowel Cancer Screening Program (NBCSP) provides biennial immunochemical faecal occult blood test (iFOBT) screening for people aged 50–74 years. Previous work has quantified the number of colorectal cancer (CRC) deaths prevented by the NBCSP and has shown that it is cost-effective. With a 40% screening participation rate, the NBCSP is currently underutilised and could be improved by increasing program participation, but the maximum appropriate level of spending on effective interventions to increase adherence has not yet been quantified.

**Objectives:**

To estimate (i) reductions in CRC cases and deaths for 2020–2040 attributable to, and (ii) the threshold for cost-effective investment (TCEI) in, effective future interventions to improve participation in the NBCSP.

**Methods:**

A comprehensive microsimulation model, *Policy1-Bowel*, was used to simulate CRC natural history and screening in Australia, considering currently reported NBCSP adherence rates, i.e. iFOBT participation (∼40%) and diagnostic colonoscopy assessment rates (∼70%). Australian residents aged 40–74 were modelled. We evaluated three scenarios: (1) diagnostic colonoscopy assessment increasing to 90%; (2) iFOBT screening participation increasing to 60% by 2020, 70% by 2030 with diagnostic assessment rates of 90%; and (3) iFOBT screening increasing to 90% by 2020 with diagnostic assessment rates of 90%. In each scenario, we estimated CRC incidence and mortality, colonoscopies, costs, and TCEI given indicative willingness-to-pay thresholds of AUD$10,000-$30,000/LYS.

**Results:**

By 2040, age-standardised CRC incidence and mortality rates could be reduced from 46.2 and 13.5 per 100,000 persons, respectively, if current participation rates continued, to (1) 44.0 and 12.7, (2) 36.8 and 8.8, and (3) 31.9 and 6.5. In Scenario 2, 23,000 lives would be saved from 2020–2040 vs current participation rates. The estimated scenario-specific TCEI (Australian dollars or AUD$/year) to invest in interventions to increase participation, given a conservative willingness-to-pay threshold of AUD$10,000/LYS, was (1) AUD$14.9M, (2) AUD$72.0M, and (3) AUD$76.5M.

**Conclusion:**

Significant investment in evidence-based interventions could be used to improve NBCSP adherence and help realise the program’s potential. Such interventions might include mass media campaigns to increase program participation, educational or awareness interventions for practitioners, and/or interventions resulting in improvements in referral pathways. Any set of interventions which achieves at least 70% iFOBT screening participation and a 90% diagnostic assessment rate while costing under AUD$72 million annually would be highly cost-effective (<AUD$10,000/LYS) and save 23,000 additional lives from 2020–2040.

## Introduction

In Australia, colorectal cancer (CRC) is the third most common cause of cancer death in men and women, with an estimated 5,597 CRC deaths and an age-standardised CRC mortality rate of 17.8 per 100,000 persons in 2019.[1] Randomised controlled trials and comprehensive modelling studies have confirmed that population screening can be a cost-effective way to improve colorectal cancer incidence and mortality rates, leading to screening related recommendations being included in CRC management guidelines internationally.[2–6] The Australian National Bowel Cancer Screening Program (NBCSP), initiated in 2006, provides free screening using an immunochemical faecal occult blood test (iFOBT).[7] Fully implemented in 2019, all Australians aged 50–74 years are invited to screen once every two years via the NBCSP.[7] According to the NBCSP Monitoring Report, over the two-year period 2015–2016, the federal and state Australian governments spent approximately AUD$56.1 million dollars on the NBCSP excluding downstream costs; this is lower than the corresponding spending on the other two government-funded population-based screening programs in Australia—BreastScreen Australia and the National Cervical Screening Program.[8] (These published cost estimates for the NBCSP should not directly be compared to those reported herein which include downstream diagnostic, surveillance and cancer treatment costs, but not all costs for program administration and overheads).

In 2015–16, around 40% of NBCSP-invited individuals participated in iFOBT screening, with an 8% positivity rate.[7] Of individuals with a positive iFOBT result, 68% had a recorded follow-up diagnostic assessment,[7] typically via colonoscopy (but with known underreporting for this rate); this is the estimated *diagnostic assessment rate*. Here, we refer to iFOBT participation and diagnostic assessment collectively as *program adherence*. Previous NBCSP evaluations, including assessments of alternative screening technologies or different target age ranges, concluded that the current NBCSP recommendations encapsulate the best option for organised CRC screening in Australia at this time, and that encouraging higher screening participation would make the program more effective whilst remaining cost-effective.[3, 4, 9–11] The currently observed participation rate for the NBCSP (~40%) is lower than rates for the other two population-based cancer screening programs in Australia; in 2014–2016, the observed 2-yearly participation was 54–55% for BreastScreen Australia and 56–58% for the National Cervical Screening Program.[12, 13] Interventions to increase screening participation through a mass-media awareness campaign have been trialed internationally,[14] and a 7-week campaign in Victoria resulted in an increase in NBCSP iFOBT kit returns to over 50%.[15, 16] It has previously been found that if NBCSP participation increased to 60% by 2020 and 70% by 2030, over 83,000 total CRC deaths would be averted in the period 2015–2040;[3] and another study found comparable improvements to NBCSP performance if participation is improved to 60% from 2020.[4] These participation increases could be achieved by mass media awareness campaigns[15, 17] or potentially via other interventions at an individual or primary care level. For example, such interventions may include changes to information provided with test kits, or educational or awareness interventions for primary care practitioners. Evaluating the cost-effectiveness of taking action to improve program participation should consider the costs associated with the intervention(s) needed to improve participation as well as additional program-related costs arising from increased participation.

With the aim of reducing the burden and impact of CRC among Australians, Health Translation South Australia’s *No Australians Dying of Bowel Cancer Initiative* was designed to develop a practical road-map and targets to eradicate CRC death in Australia. There are four target areas: (1) lifestyle change, (2) NBCSP participation, (3) access to equitable, excellent and affordable colonoscopy, and (4) improved survival for patients with advanced CRC. To support the *No Australians Dying of Bowel Cancer Initiative*, the current study aims to evaluate the expected outcomes of increases to iFOBT participation and diagnostic assessment rates. These hypothetical improvements to screening participation were evaluated in terms of the potential improvements to CRC incidence and mortality, and the maximum investment which could be made in achieving these improvements while remaining cost-effective (the threshold for cost-effective investment, or TCEI) was also calculated.

## Methods

### *Policy1-Bowel* model

A comprehensive microsimulation model, *Policy1-Bowel*, was used for the evaluation. *Policy1-Bowel* has been adapted from the Dutch Adenoma and Serrated pathway to Colorectal Cancer model (ASCCa),[18] and simulates both the adenoma-carcinoma pathway and serrated pathway in CRC development. The location, size, shape, dysplasia, and architecture of conventional adenomas, and the location and size of sessile serrated polyps (sometime referred to as sessile serrated adenoma), are modelled. A multi-cohort simulation with 2 million males and 2 million females per birth cohort between 1911 and 2010 was completed, with individuals simulated from age 20 years to age 89 years or death. The model has been extensively calibrated and validated to published natural history data and CRC-related data observed in the Australian setting, as previously described in detail.[3] A summary of the key input parameters used by *Policy1-Bowel* is provided in Table 1.

**Table 1:** Key model parameters used by *Policy1-Bowel*. All costs are in 2018 Australian dollars (AUD).

Key model parameter	Value	Reference
**Cost**		
Postage (one-way)	$2	Based on values used in [3]
Test kit sent	$8	Based on values used in [3]
Test kit received and analysed in the lab	$20	Based on values used in [3]
GP consultation for FOBT positive result	$37.60	MBS item 23 [19]
Colonoscopy, with/without polypectomy (without complication)	$1,800	Based on values used in [3]
Colonoscopy with/without polypectomy (with complication)	$17,351	Inflated cost of DRG-AG item G48A[20] based on CPI in health from June 2011 to June 2018[21]
Stage 1 cancer treatment	$46,531	Value from Ananda et al. [22] based on CPI in health from June 2011 to June 2018[21]
Stage 2 cancer treatment	$74,311	
Stage 3 cancer treatment	$110,009	
Stage 4 cancer treatment	$96,426	
**iFOBT test characteristics (per person)**		
Specificity for any adenoma	94.8%	Obtained via calibrating to iFOBT positivity rates observed in NBCSP and colonoscopy outcomes among positive iFOBT [23]
Sensitivity for conventional adenoma of any size	15.2%	
Sensitivity for conventional adenoma > 5mm	30.2%	
Sensitivity for conventional adenoma >10mm	41.5%	
Sensitivity for CRC	58.6%	
**Colonoscopy test detection rate (per lesion)**		
Conventional adenoma 1–5 mm	79%	Van Rijn et al 2006 [24, 25]
Conventional adenoma 6–9 mm	85%	
Conventional adenoma ≥10mm	92%	
Sessile serrated polyps (any size)	78%	
CRC (any stage)	95%	
**Colonoscopy completion rate**	100% to the end of cecum	Based on values used in [3]
**Colonoscopy adverse event probability**		
Non-fatal adverse event	0.27%	AIHW 2015 [23, 26]
Death	0%	AIHW 2015 [23, 26], Jentschura et al 1994 [27]
**Baseline colonoscopy compliance rate**		
Follow-up colonoscopy	71%	AIHW 2015 [23]
Surveillance colonoscopy	80%	Based on values used in [3]
**5-year survival rate in patient detected with colorectal cancer due to symptoms shown**		
Stage 1 cancer	86.9%	Morris et al 2007 [28]
Stage 2 cancer	73.0%	
Stage 3 cancer	42.4%	
Stage 4 cancer	9.5%	
**Relative 5-year survival of screen-detected cancer versus symptomatically-detected cancer**		
Stage 1 cancer	1.1	Parente et al 2015, Gill et al 2014, Pande et al 2013 [29–31]
Stage 2 cancer	1.2	
Stage 3 cancer	1.4	
Stage 4 cancer	2.3	

### Program adherence scenarios

The modelled comparator for the study was the existing NBCSP with a 40% iFOBT screening participation rate for eligible individuals and 70% diagnostic assessment rate for individuals with a positive iFOBT result, based on data observed in 2006–2016.[3] A schematic of the modelled screening and diagnostic pathway is provided in Appendix A of S1 File. Three scenarios assuming incremental improvements to program adherence were simulated, to reflect the possible impact of initiatives aimed at increasing adherence. Scenario 1 assumes that only the diagnostic assessment rate is improved. Scenario 2 models improved iFOBT screening participation rates to 60% in 2020, subsequently increasing to 70% by 2030, rates likely to be feasible based on the findings of a recent Victorian mass-media campaign,[15, 16] as well as an improved diagnostic assessment rate. Scenario 3 is an optimistic goal which assumes very high program adherence at 90% (i.e. 90% screening participation and diagnostic assessment). It should be noted that the 90% adherence assumption is intended as a proxy for perfect adherence in this evaluation, as it is inappropriate for some individuals to undergo iFOBT screening and/or colonoscopy assessment. The scenarios are summarised in Table 2. Colonoscopy surveillance for management of individuals with previous removal of an adenoma was modelled based on current guidelines, at 80% compliance in all scenarios.[32, 33]

**Table 2:** Screening participation rate and diagnostic assessment compliance rate assumptions for all modelled scenarios.

Scenario name	Modelled overall NBCSP participation rates ^*a*^^,^^*b*^			Modelled diagnostic assessment compliance rates
	Prior to 2020	2020–2029	2030 onwards	
**Comparator**	33–40% in 2006–2015, ∼40% from 2016 onwards^*c*^	∼40%	∼40%	∼70% at all times^*d*^
**Scenario 1**	As per the comparator	As per the comparator	As per the comparator	∼70% in 2006–2019, 90% from 2020 onwards
**Scenario 2**	As per the comparator	60%, gradually increasing to 70%	70%	∼70% in 2006–2019, 90% from 2020 onwards
**Scenario 3**	As per the comparator	90%	90%	∼70% in 2006–2019, 90% from 2020 onwards

NBCSP–National Bowel Cancer Screening Program

^a^ The modelled screening participation and diagnostic assessment rates shown in Table 2 are overall rates in the Australian population; age- and sex-specific rates derived from the currently observed rates were considered in the model.

^b^ The model takes into account the phased implementation of the NBCSP in 2006–2018 when simulating the NBCSP, with full implementation from 2019.(7)

^c^ The modelled 33–40% screening participation rates in 2006–2015 were based on the observed NBCSP iFOBT screening participation rate.(7) Screening participation rates modelled for 2017 onwards were extrapolated from data observed in 2006–2016.

^d^ The modelled ~70% NBCSP diagnostic assessment compliance rates were based on the reported NBCSP diagnostic assessment compliance rates in 2015–16.(7)

A common question raised by health and governmental bodies is whether complete or near-complete eradication of CRC could be achieved via population-level screening. To address this, we also performed a supplementary evaluation of an extreme scenario which would reflect the maximum number of CRC deaths which could potentially be prevented via population-level screening. This scenario assumed supplementary screening colonoscopies were offered to people aged 40 and 60 years in parallel to the current NBCSP, with both the NBCSP and colonoscopy assumed to have a 90% participation rate. As this is outside of the modality of the current NBCSP and not a focus of this paper, details of this scenario are included in Appendix C of S1 File.

### Cost and test characteristics

A health services perspective was used in our analysis. We included costs associated with: sending iFOBT kits to eligible individuals; laboratory analysis of the completed iFOBT samples; GP visits for follow-up of positive iFOBT results; colonoscopy procedures (with/without polypectomy and/or adverse events) to follow-up positive iFOBT result and for surveillance purposes; and CRC treatment. Costs associated with existing NBCSP participation promotion, individuals’ out-of-pocket costs and administration-related costs (other than the costs of sending iFOBT kits) were not included. The modelled test characteristics of iFOBT and colonoscopy were informed by a review of the international literature, calibrated to the outcomes observed in the NBCSP.[3] Key modelled cost and test characteristics of iFOBT and colonoscopy are provided in Table 1. All costs are presented in 2018 Australian dollars.

### Modelled analysis

The 2000–2013 Australian population and projected 2014–2040 populations estimated by the Australian Bureau of Statistics (ABS) were used to calculate the population-level results.[34, 35] The 2001 Australian Population was used to calculate the age standardised rates (ASRs).[35] The modelled output includes age-specific CRC incidence and mortality rates, costs, and number of screening and diagnostic tests for 2006–2040. The number-needed-to-colonoscope (NNC) per CRC death prevented for each scenario was calculated by dividing the number of additional colonoscopies by the number of deaths prevented in 2020–2040 versus the comparator (i.e. an estimate of the NNC over this time period; this cannot be directly compared with calculations over the lifetime of a specific birth cohort).

Additionally, single-age cohorts of 100 million males and 100 million females who would be invited for the full screening program in all modelled scenarios were simulated to calculate cost-effectiveness. Costs and life-years were calculated over the lifetime of the single birth cohort and discounted at a rate of 5% per annum[36] starting at age 40 years (the first year of intervention considered in any main or supplementary scenario in this analysis). The cost-effectiveness ratio (CER) versus the comparator (the NBCSP at previously observed adherence rates) was calculated for all scenarios using the additional discounted lifetime costs and additional discounted life-years saved (LYS). The CER calculation does not include the direct or overhead costs associated with the delivery any of the interventions or combination of interventions which would likely be required to achieve this increase in program adherence; rather, the computation of the TCEI below is designed to provide a threshold for these costs.

For each scenario evaluated in this study, the *threshold for cost-effective investment* (TCEI) to improve program adherence was calculated; this is defined as the additional discounted cost at which the scenario’s cost-effectiveness ratio reaches a reference threshold, accounting for increases or decreases to costs such as additional iFOBT tests, program-related colonoscopies, and cancer treatment. Indicative WTP thresholds of AUD$30,000–50,000/LYS for prevention evaluations, including cancer screening interventions, have been used by previous studies.[37–40] In the current study, a conservative approach was taken to estimate the TCEI using a previously used indicative WTP threshold of $30,000/LYS and two alternate lower WTP thresholds of AUD$10,000/LYS and AUD$20,000/LYS. The TCEI was calculated for individuals as a lifetime discounted cost (from age 20 to 99 or death, whichever occurs first) and then converted to an indicative annualised national cost, to estimate a maximum allowable investment on increasing program adherence while remaining cost-effective. The TCEI for an individual is a total cost over their lifetime, without specifying when these costs occur. This could be any combination of undiscounted costs that occur at or before the age of 40 years (at a discount rate of zero) and discounted costs that occur later in life. To approximate a threshold for annual national investment in Australia, we then multiplied the TCEI for an individual by the number of individuals aged 50 years in 2020 predicted by the ABS. Sensitivity analyses on the estimated TCEI assuming more and less aggressive pre-cancer natural history pathways were performed; see Appendix B of S1 File for detailed natural history assumptions. This analysis was included to reflect uncertainties inherent in the *Policy1-Bowel* model.

## Results

### Health outcomes

**Fig 1:**
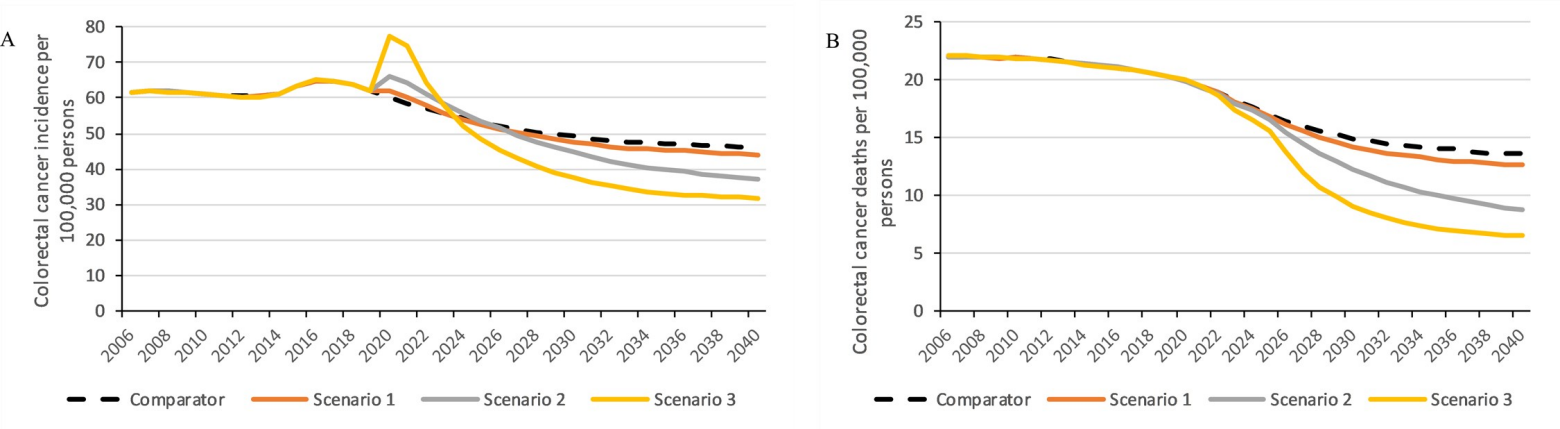
Estimated age-standardised rate (ASR) of (a) colorectal cancer incidence and (b) colorectal cancer mortality from 2006 to 2040 per 100,000 persons. Rates standardized to the 2001 Australian Standard Population.

**Fig 2:**
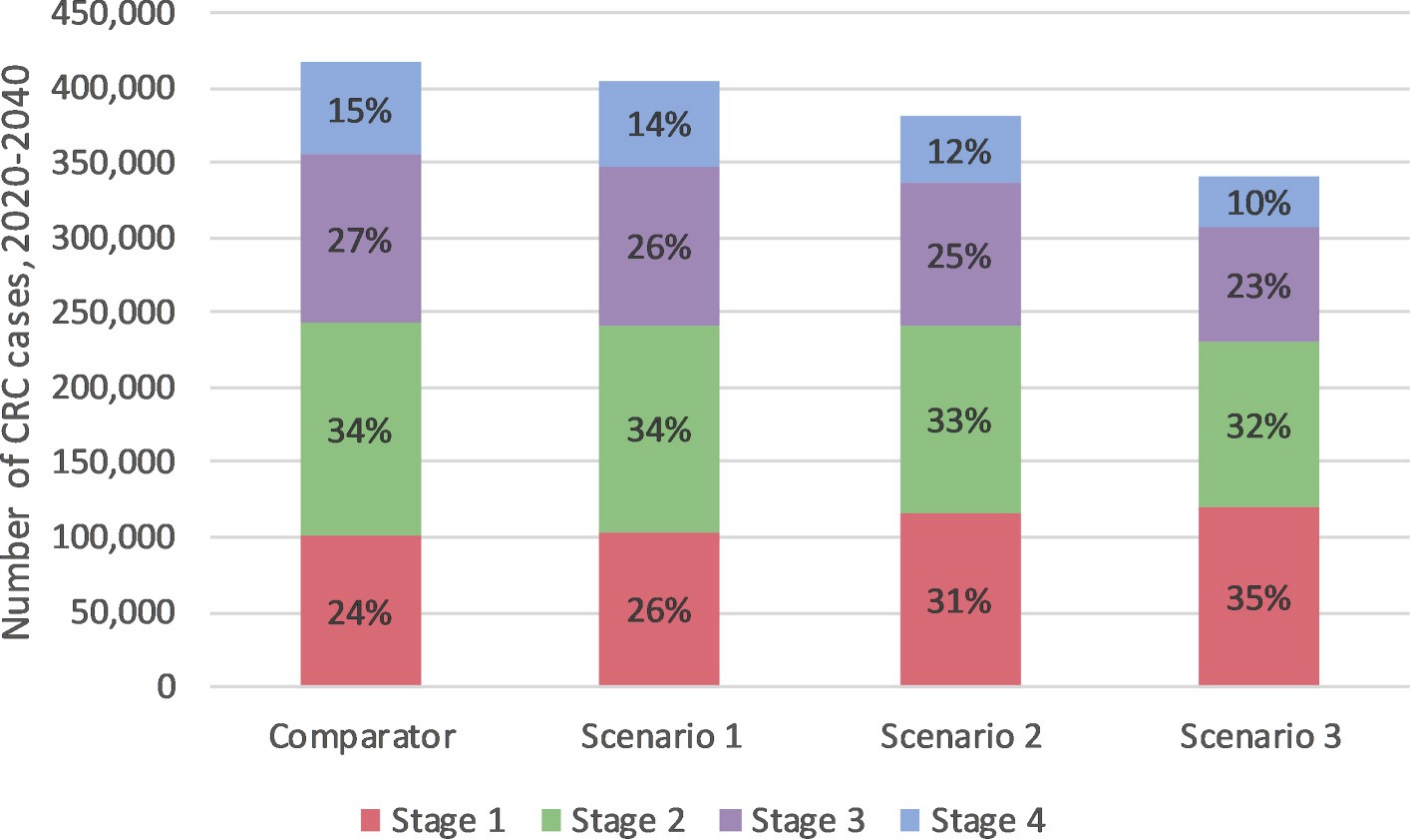
Estimated total number of incident colorectal cancer cases in the period 2020–2040 by stage. For each scenario, the proportion of cases diagnosed at each stage is labelled in brackets.

Compared to currently observed program adherence, increasing diagnostic assessment rates and/or iFOBT participation could prevent an additional 10,900–75,200 CRC cases and 5,700–42,700 CRC deaths in 2020–2040 (Table 3). By 2040, CRC incidence and mortality ASRs per 100,000 were predicted to be reduced from 46.2 and 13.6 respectively if current program adherence continues (comparator) to 44.0 and 12.7 if diagnostic assessment rates increased to 90% (Scenario 1), 36.8 and 8.8 if iFOBT participation rates increased to 60–70% and diagnostic assessment rates increased to 90% (Scenario 2), and 31.9 and 6.5 if overall program adherence increased to 90% (Scenario 3). The impact on these rates is illustrated in Fig 1. Improvements to screening participation led to a higher proportion of incident CRCs being detected at an earlier stages as shown in Fig 2, thus improving overall survival.

**Table 3:** Estimated colorectal cancer cases and deaths, number of colonoscopy assessments, colonoscopy-related adverse events, and direct program-related costsover the period 2020–2040 in the Australian population (number of cases, deaths, colonoscopies and adverse events rounded to nearest hundred).

	*Colorectal cancer cases*		*Colorectal cancer deaths*		*Number of colonoscopies* ^*a*^		*Colonoscopy-related adverse events*		*Total undiscounted costs*^*b*^ *(AUD billion)*		Number needed to colonoscope (NNC) per CRC death prevented
	Total 2020–2040	vs Comparator (% change)	Total 2020–2040	vs Comparator (% change)	Total 2020–2040	vs Comparator (% change)	Total 2020–2040	vs Comparator (% change)	Total 2020–2040	vs Comparator (% change)	
**Comparator**	416,500	N/A	131,500	N/A	2,970,600	N/A	8,000	N/A	$40.79	N/A	N/A
**Scenario 1**	405,500	-10,900 (-2.6%)	125,800	-5,700 (-4.3%)	3,534,400	563,800 (18.7%)	9,500	1,500 (18.7%)	$40.68	-$0.11 (-0.3%)	98.6
**Scenario 2**	381,000	-35,500 (-8.5%)	108,500	-23,000 (-17.5%)	5,275,100	2,304,500 (77.3%)	14,200	6,200 (77.3%)	$41.53	$0.74 (1.8%)	100.0
**Scenario 3**	341,200	-75,200 (-18.1%)	88,800	-42,700 (-32.5%)	6,883,700	3,913,100 (132.5%)	18,600	10,600 (132.5%) dfasfdsa12323213(132(132.2%)	$41.09	$0.30 (0.7%)	91.5

N/A- not applicable

^a^ Includes both colonoscopy assessments performed to follow-up individuals with positive iFOBT results and colonoscopies to provide surveillance for individuals with the removal of at least one adenoma and/or sessile serrated polyps. Out-of-program colonoscopies are not included in the model estimates.

^b^Costs considered are the undiscounted costs associated with sending the iFOBT kits, laboratory analysis of the completed iFOBT samples, general practitioner visit for follow-up of positive iFOBT results, colonoscopy procedures with/without adverse events (and polypectomy if required) to follow-up positive iFOBT result and to provide surveillance, and colorectal cancer treatments.

### Costs and resource utilisation

**Fig 3:**
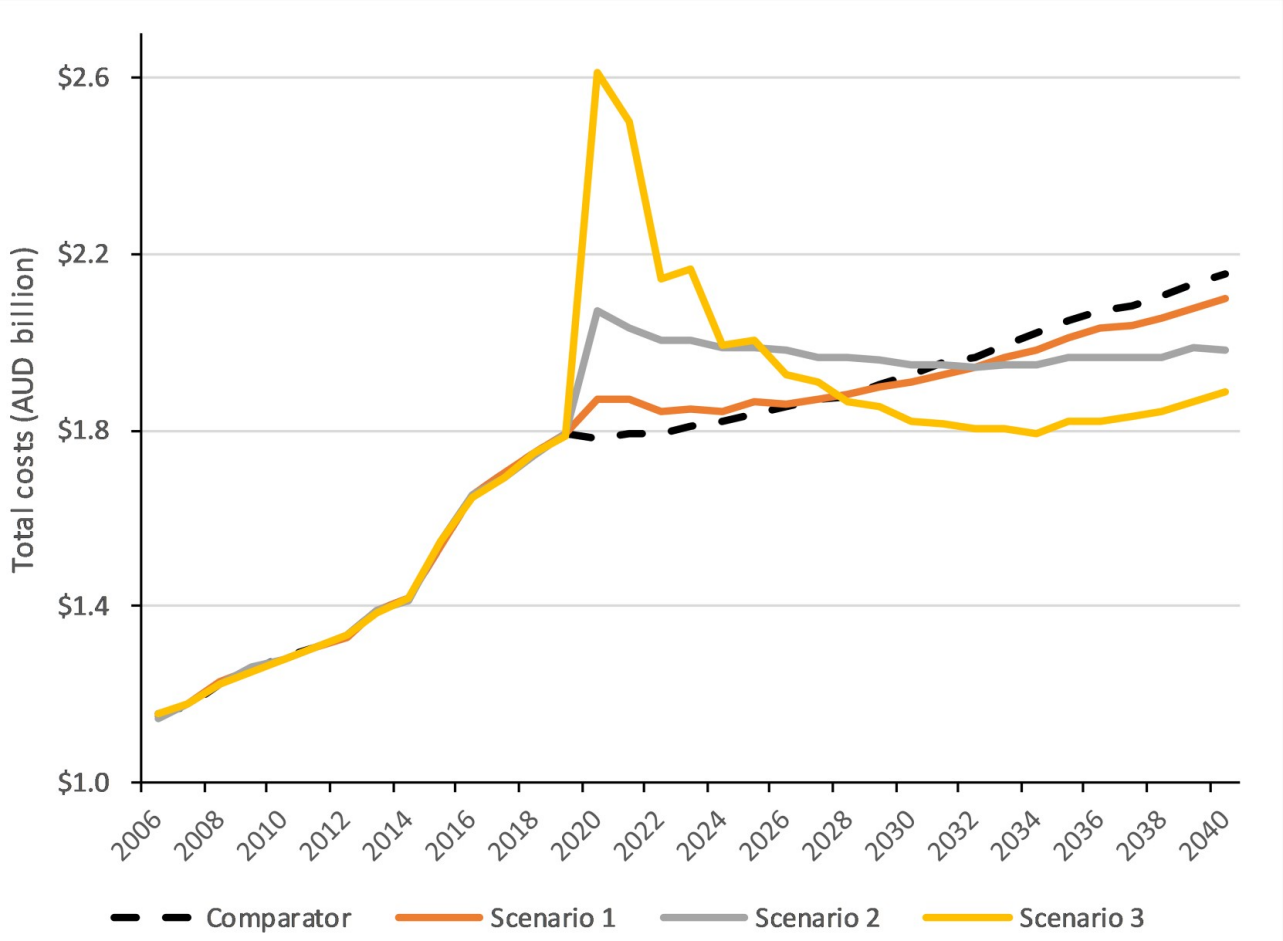
Estimated undiscounted costs to provide iFOBT screening, colonoscopy follow-up and surveillance, and colorectal cancer treatment in 2006–2040.

Increasing NBCSP program adherence would lead to a short-term increase in annual expenditure to provide additional iFOBT screening, diagnosis, surveillance, and CRC treatment, leading to higher annual costs than the comparator as shown in Fig 3. After 2030, annual costs for all scenarios would be lower than the comparator, which can be attributed to CRC treatment averted and reduced costs due to cases diagnosed at an earlier stage. As program adherence is increased, both the total number of CRC cases and the proportion of cases in later stages decreases, translating to better overall survival and lower costs. The total undiscounted expenditure in 2020–2040 across scenarios was estimated to be AUD$40.68–41.09 billion, vs AUD$40.79 billion for the comparator (Table 3).

The number of colonoscopies between 2020–2040 was predicted to increase by 19–132% across scenarios, equivalent to 26,800–186,300 additional colonoscopies annually; the associated NNC was 98.6–100.0 per CRC death prevented.

### Threshold analysis for cost-effective investment (TCEI)

The TCEI for interventions to improve program adherence while remaining under indicative WTP thresholds of AUD$10,000/LYS, AUD$20,000/LYS and AUD$30,000/LYS were calculated (Table 4). The annual national TCEI in 2020, considering the range of alternate thresholds, was estimated to be AUD$14.9–30.5 million in Scenario 1, AUD$71.9–168.4 million in Scenario 2, and AUD$76.49–194.5 million in Scenario 3. The discounted TCEI per person over their lifetime was estimated to be AUD$44–91, AUD$214–502, and AUD$228–589 for Scenarios 1–3, respectively (Table 4).

**Table 4:** Estimated life-years, lifetime cost, and cost-effectiveness ratio (CER) vs current screening rates for a single birth cohort, as well as estimated thresholds for additional investment while remaining cost-effective (TCEI) for each scenario while remaining under willingness-to-pay thresholds of $10,000/LYS, $20,000/LYS, and $30,000/LYS. The total TCEI for Australia is an annualized estimate for the 2020 population. All costs are in 2018 Australian dollars.

	Undiscounted		Discounted			Maximum investment per person under WTP threshold (AUD) ^*d*^			Maximum investment per year in Australia under WTP threshold (AUD) ^*e*^		
	Life-years per person	Lifetime cost per person	Life-years per person ^*a*^	Lifetime cost per person	Interim ICER ^*b*^	$10,000/LYS	$20,000/LYS	$30,000/LYS	$10,000/LYS	$20,000/LYS	$30,000/LYS
**Comparator**	60.847	$7,711	37.412	$1,827	-	-	-	-	-	-	-
**Scenario 1**	60.862	$7,432	37.415	$1,805	Cost-saving ^*c*^	$44.47	$67.65	$90.84	$14.92 million	$22.7 million	$30.47 million
**Scenario 2**	60.931	$6,489	37.427	$1,756	Cost-saving ^*c*^	$214.39	$358.15	$501.90	$71.92 million	$120.15 million	$168.37 million
**Scenario 3**	60.951	$6,280	37.430	$1,779	Cost-saving ^*c*^	$228.01	$408.32	$588.63	$76.49 million	$136.98 million	$197.47 million

Life-years have been presented to 3 decimal places; costs have been rounded to the nearest dollar, and TCEI values are presented to two decimal places.

^a^ Discounted life-years per person and discounted costs are calculated with a 5% discount rate per year. The cost-effectiveness ratio (CER) is then calculated as the additional cost divided by the additional life-years vs the comparator.

^b^ Without considering the direct or overhead costs of interventions needed to increase participation/adherence.

^c^ All scenarios are cost-saving and more effective vs the comparator (see footnote b for interpretation; does not account for additional intervention costs).

^d^ Lifetime undiscounted cost.

^e^ Assuming 2020 Australian population.(34) For computational purposes, the theoretical investment in interventions to increase adherence for calculating the TCEI are assumed to be incurred at age 50.

### Supplementary and sensitivity analysis

In the supplementary analysis, the addition of screening colonoscopies at ages 40 and 60 (an extreme scenario) would reduce CRC incidence and mortality ASRs to 21.2 and 4.1 per 100,000 respectively by 2040. However, it would also be associated with a very large increase in colonoscopy demand (615% increase over 2020–2040 vs the comparator), and would not be cost-effective, even when considering only the direct costs of the additional colonoscopies involved and without considering any overhead or establishment costs of interventions needed to achieve high adherence (see Appendix C of S1 File for more details).

In the sensitivity analyses, when assuming a more aggressive natural history, the TCEIs were estimated to increase, ranging from AUD$18.6–38.6 million, AUD$105.0–240.0 million, and AUD$116.1–282.9 million, respectively per year nationally to remain under indicative WTP thresholds of AUD$10,000–30,000/LYS. With a less aggressive natural history these TCEIs decrease to AUD$11.4–25.0 million, AUD$51.9–136.1 million, and AUD$53.7–162.0 million respectively. These ranges of thresholds reflect uncertainties in the natural history of colorectal cancer. Detailed results are included in Appendix D of S1 File.

## Discussion

To our knowledge, this is the first evaluation in the Australian context to consider the health and economic impact of increases in diagnostic assessment rates alongside increases to iFOBT participation, illustrating the importance of both components of program adherence in achieving optimal health outcomes for the NBCSP. Our results indicate that improving program adherence could further reduce CRC incidence and mortality by up to 18% and 33% respectively in the period 2020–2040. The TCEIs calculated are a new finding in relation to the NBCSP and demonstrate that considerable additional investment could be made in improving program adherence, whilst still maintaining cost-effectiveness in terms of the overall spend in relation to bowel cancer screening. We found that investment of up to AUD$72M per annum nationally (with a range of AUD$51-105M in sensitivity analysis), beyond current and previous investments, could be justified if it is spent on effective interventions which increase iFOBT participation to 70% and diagnostic assessment rates to 90%. The Australian Institute of Health and Welfare reported that total health expenditure per person for one year was over AUD$7,400 in Australia in the 2016–17 financial year.[41] The estimated TCEI per person suggests than an additional AUD$214–501 per person over their lifetime could be spent to increase the likelihood of screening to 70% and remain cost-effective.

This study found that improving iFOBT screening to 60% from 2020 and 70% by 2030 while increasing diagnostic assessment rates to 90% would prevent nearly 23,000 additional CRC deaths over the period 2020–2040 versus the number that would be prevented at current participation rates. This is similar to our previous findings,[3] which found that approximately 25,000 additional deaths could be prevented from 2020–2040 if iFOBT screening were gradually increased to 60% by 2020 and 70% by 2030 without any increase to diagnostic assessment rates (see Scenario 3 versus Scenario 1 in Lew et al [3]). The supplementary analysis, which considered the addition of screening colonoscopies at ages 40 and 60, evaluated the limits of screening in reducing CRC death rates in Australia. Although this is an extreme scenario which would dramatically reduce CRC deaths, it would not eradicate CRC entirely, and would come at very high costs and resource demand.

A strength of this study is the use of a comprehensive and well-calibrated model[3, 9] to address scenarios for program adherence, including both realistic goals in the Australian setting and high participation scenarios to assess the limits of screening. The model incorporates detailed screening, diagnosis and surveillance management pathways, observed NBCSP adherence rates, and cancer treatment costs consistent with the best available data. As with all models, some parameters are based on assumptions where real-world data are unavailable, and available data may be uncertain. This has been addressed in part via sensitivity analyses considering more and less aggressive precancer natural history parameter sets, which reflect uncertainties in the model. Future program adherence for the comparator is based on extrapolations from current observations, and there is uncertainty around current participation observations, particularly in terms of the diagnostic assessment rate. The diagnostic colonoscopy assessment rate assumed for the comparator is based on the current reported Australian rate, which is likely to be an underestimate due to non-mandatory reporting of colonoscopy despite it being a program performance indicator.[7] Therefore the potential health benefits, cost-effectiveness and TCEI associated with improved diagnostic assessment rates may have been overestimated. Studies have shown that CRC treatment costs have been rapidly increasing over the past 10 years.[22] The CER and TCEI associated with improved program adherence may change if CRC treatment costs continue to increase, and is also dependent on other factors associated with screening. However, in considering these issues, we took into account a wide range of assumptions for future program participation in our analysis, and our main findings are based on conservative choice of the indicative willingness-to-pay threshold. This increases the robustness of our main finding—that it would be very cost-effective to spend an additional AUD$70M or more on interventions which achieve both a 60% program screening participation by 2020, 70% by 2030, and a 90% diagnostic assessment rate from 2020 onwards.

Our study has highlighted the importance of diagnostic assessment rates and their impact on NBCSP outcomes. Additionally, ensuring colonoscopy use is appropriate and timely is critical for CRC control. Our study indicates that performing population-level screening colonoscopy at 40 and 60 years as an adjunct to existing NBCSP iFOBT screening is not optimal for CRC prevention in the general population. Currently, of the approximately one million colonoscopies occurring annually in Australia about 3% were estimated to be attributable to the follow-up individuals with positive NBCSP iFOBT results, including NBCSP related surveillance.[42] Efforts should be made to reduce non-guideline recommended colonoscopy use by encouraging eligible individuals into iFOBT screening.

Lack of understanding of asymptomatic CRC and awareness of screening tests have been identified as barriers to NBCSP participation.[43] Interventions to improve adherence such as general public awareness campaigns, health professional endorsement, and non-responder follow-up have been trialed.[14, 15, 44, 45] GP perceptions of iFOBT screening are not always aligned with evidence of its effectiveness, suggesting that they may not be promoting program adherence.[46] Raising awareness in both the general population and in primary care would help improve program adherence, especially around diagnostic assessment. A considerable number of lives and potential long-term savings to government expenditure could be achieved by improving NBCSP adherence even with targeted and low-cost interventions and campaigns.[47]

The findings of this paper complement a recently published study by some of us.[48] The prior study involved evaluation of the cost-effectiveness of a seven-week mass media campaign run in the state of Victoria in Australia in 2017; this campaign increased iFOBT screening participation from 42.7% to 57.2% at a cost of AUD$1.06 million.[15, 16, 48] The campaign was found to be highly cost-effective, with a cost-effectiveness ratio of AUD$2,470/LYS. We also predicted that an extended national campaign would be highly cost-effective and this prediction helped underpin the case for investment in a AUD$10M mass media campaign currently being run in Australia. That work took the opposite approach to the methodology of the current paper since it used real-world data to inform the costs and impact on participation from a campaign, and then calculated the cost-effectiveness. The analysis presented in this paper, which calculates a maximum potential cost (TCEI) for any intervention which improves program participation, is complementary as it provides guidance for appropriate cost limits and effectiveness requirements for new interventions.

The findings presented in this study suggest that investments should continue and could be extended to interventions targeted at improving follow-up adherence. Campaigns targeting population subgroups with lower screening rates and/or higher risk could potentially be more effective and cost-effective.[49] Alongside screening, primary prevention is also a critical target area for intervention, as almost half of CRC cases in Australia are attributable to known modifiable risk factors.[50, 51] To move towards the possibility of ‘CRC elimination’, as is now being proposed for cervical cancer (considering a potential elimination threshold of 4 cases per 100,000 women),[52] the successful implementation of a range of cost-effective strategies would likely be required over the longer term, potentially including primary prevention activities, further improvements in screening technologies, increased NBCSP screening and diagnostic assessment adherence (as considered here), and colorectal cancer treatment and survival improvements. We have estimated that the Australian government is estimated to spend more than AUD$1 billion annually to provide iFOBT screening, diagnostic assessment to follow-up positive iFOBT results and CRC treatment,[3] This estimated annual expenditure is broadly consistent with another Australian study.[22] In the current study we found that interventions costing less than AUD$72 million a year in total which improved NBCSP participation to 60–70% and diagnostic assessment rates to 90% would be highly cost-effective and could further reduce the burden of CRC in Australia. This finding could inform resource planning and policy decision making towards maximising the impact of the NBCSP.

Internationally, the design and implementation of colorectal cancer screening varies widely between regions, including the targeted groups, test technology used, and delivery mechanisms.[53] As population-based CRC screening becomes utilized in more countries, ensuring programs are used to their full potential will be increasingly important. Although the results here may not be directly applicable to international settings, the methodologies of this study, including the calculation of the TCEI for guiding funding, should be helpful for screening programs internationally.

## Conclusion

CRC incidence and mortality could be reduced to 36.8 and 8.8 respectively per 100,000 persons by 2040 if iFOBT participation can be increased to 60% by 2020 and 70% by 2030, and confirmed diagnostic assessment rates can be increased to 90%. In achieving this, the number of Australians dying of CRC annually would be reduced by 2,400 (from 6,600 at current program adherence to 4,200) in 2040. Any investment in a combination of effective interventions which achieves these targets while costing under AUD$72 million per annum would save lives and be highly cost-effective.

## Supporting information

S1 File Improving Australian National Bowel Cancer Screening Program outcomes through increased participation and cost-effective investment–Technical Appendix. (DOCX)

## Abbreviations

ASR: age-standardised rate
CER: cost-effectiveness ratio
CRC: colorectal cancer
iFOBT: immunochemical faecal occult blood test
LYS: life-years saved
NBCSP: Australian National Bowel Cancer Screening Program
NNC: number-needed-to-colonoscope
WTP: willingness-to-pay
TCEI: threshold for cost-effective investment
