# Supplementary material for: Improving Australian National Bowel Cancer Screening Program outcomes through increased participation and cost-effective investment
Source: PLoS One. 2020 Feb 3;15(2):e0227899. doi: 10.1371/journal.pone.0227899 (PMC6996821; doi:10.1371/journal.pone.0227899)
Supplement: S1 File — (DOCX) [file pone.0227899.s001.docx]

Improving Australian National Bowel Cancer Screening Program outcomes through increased participation and cost-effective investment – Technical Appendix

# Appendix A – *Policy1-Bowel* Natural History Diagram


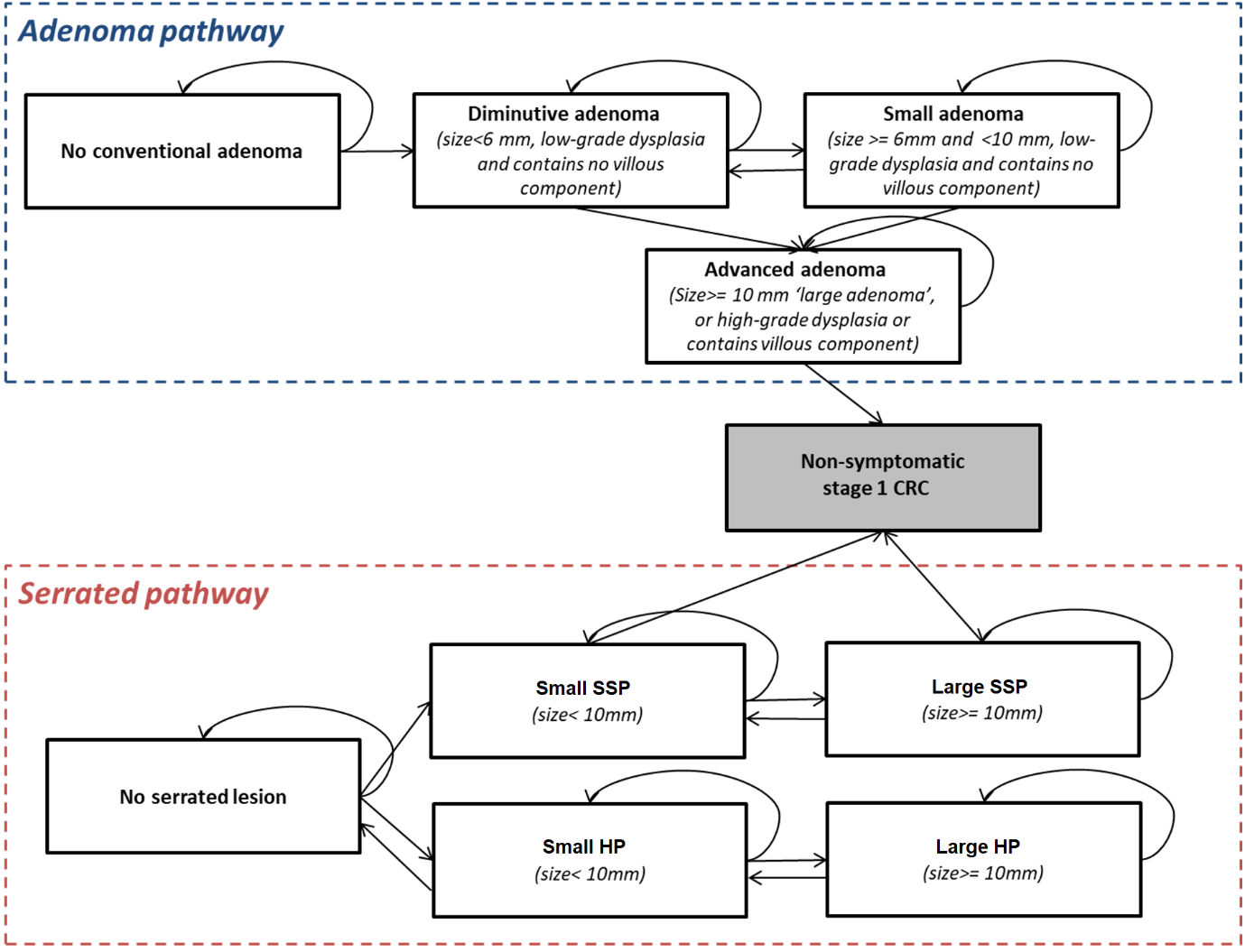


Figure A1 – Modelled precancer natural history pathway in *Policy1-Bowel*. HP – hyperplastic polyp; SSP – sessile serrated polyp (also known as sessile serrated adenoma).


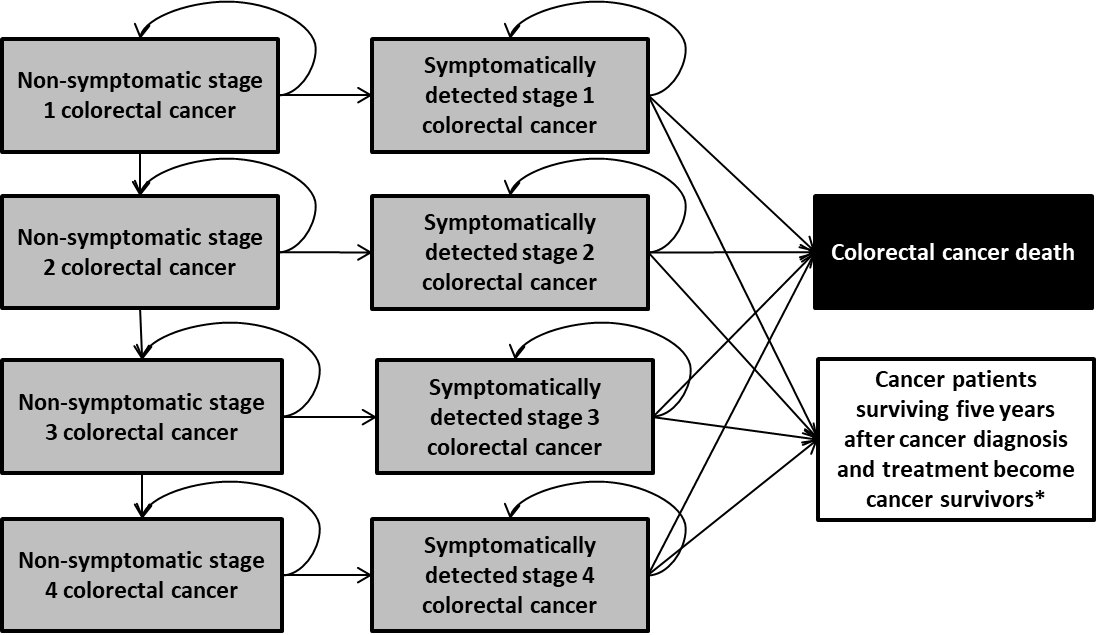


Figure A2 – Modelled colorectal cancer natural history in *Policy1-Bowel*.

** Cancer patients surviving five years after diagnosis and treatment become cancer survivors. Cancer survivors in the model were assumed to have no additional risk of death due to colorectal cancer compared with the average population with no colorectal cancer.*


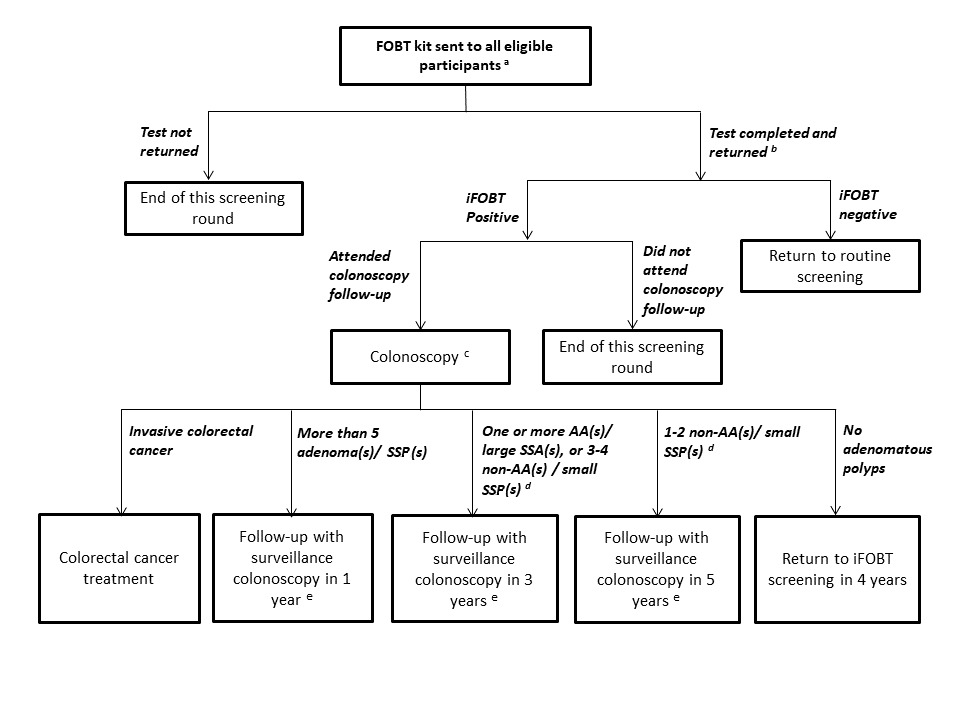


Figure A3 – Modelled NBCSP screening management.

*AA- advanced adenoma; SSP – sessile serrated polyp;*

*^a^ iFOBT kits were assumed to be sent to all individuals at eligible ages regardless of whether individuals were under surveillance colonoscopy management due to a previous positive colonoscopy finding.*

*^b^ Among the returned kits, about 1.6% were assumed to be incorrectly completed; a new kit would be sent out to these cases for re-testing and the second test was assumed to always be correctly completed.(1)*

*^c^ Polypectomy was assumed to be performed on all detected adenomatous polyps.*

*^d^ Advanced adenoma are adenomas with large size or possessing characteristics of tubular villous/villous growth pattern and/or high-grade dysplasia.*

*^e^ Management for individuals who attend colonoscopy surveillance was modelled based on guideline recommendations and expert consultation (for management not specified by the guideline).*

# Appendix B - Summary of key model parameters

## Precancer natural history

The modelled precancer natural history assumptions were systematically calibrated to the data observed in COCOS trial (a Dutch trial of colorectal screening with colonoscopy and CT

colonography).[1] The calibration targets data including age- , sex- and size- specific prevalence of adenoma, advanced adenoma, and serrated lesions, proportion of adenoma by degree of dysplasia proportion of adenoma by degree of villosity, the distribution of adenoma multiplicity, advanced adenoma multiplicity and serrated lesion multiplicity among individuals detected with bowel polyps. More than 800,000 scenarios with different natural history assumptions were assessed. The least-squares method was used to examine the goodness-of-fit of the natural history solutions. Detailed model calibration outcomes have been reported elsewhere.[2] The set of natural history assumptions which best fitted to the COCOS data was selected for base case analysis; the natural history sets that predicted the highest colorectal cancer incidence/mortality rate among the top 200 best fitted natural history sets were selected for alternative natural history assumptions for uncertainty analysis (Table B2). Figure B1 compares the modelled age-specific colorectal cancer incidence and mortality rates associated with the base case and the alternative precancer natural history assumptions evaluated in the sensitivity analysis. The age-standardised colorectal cancer incidence rate and colorectal cancer mortality rate per 100,000 persons associated was 62.5 and 23.1, respectively when assuming the base case natural history assumptions. The corresponding rates were 53.2 and 19.6 respectively when assuming the least aggressive natural history assumptions, and 75.1 and 28.7 respectively when assuming the most aggressive natural history assumptions.

Table B2 - Summary of modelled natural history parameters of the conventional adenoma pathway and serrated polyp pathway of colorectal cancer development.

| **Parameters** | | **Baseline** | **Most aggressive**  **precancer natural history** | **Least aggressive**  **precancer natural history** |
| --- | --- | --- | --- | --- |
| **Conventional adenoma-carcinoma Pathways** | | | | |
| *Adenoma incidence rate ^a^* | | | | |
|  | Male, 20-39 years | 0.01% | 0.03% | 0.01% |
|  | Male, 40-49 years | 0.07% | 0.09% | 0.05% |
|  | Male, 50-54 years | 0.10% | 0.18% | 0.11% |
|  | Male, 55-59 years | 0.20% | 0.25% | 0.17% |
|  | Male, 60-64 years | 0.25% | 0.32% | 0.21% |
|  | Male, 65-69 years | 0.28% | 0.34% | 0.23% |
|  | Male, 70-94 years | 0.31% | 0.35% | 0.25% |
|  | Male, 75+ years | 0.34% | 0.38% | 0.27% |
|  | Female, 20-39 years | 0.00% | 0.02% | 0.01% |
|  | Female, 40-49 years | 0.05% | 0.07% | 0.04% |
|  | Female, 50-54 years | 0.07% | 0.14% | 0.08% |
|  | Female, 55-59 years | 0.14% | 0.20% | 0.11% |
|  | Female, 60-64 years | 0.18% | 0.25% | 0.15% |
|  | Female, 65-69 years | 0.20% | 0.26% | 0.16% |
|  | Female, 70-94 years | 0.22% | 0.28% | 0.18% |
|  | Female, 75+ years | 0.24% | 0.29% | 0.19% |
| *Adenoma size progression and regression rate ^a^* | | | | |
|  | Progress from diminutive (<6 mm) to small (6-9 mm) size | 10% | 17% | 10% |
|  | Progress from small (6-9 mm) to large (>=10 mm) size | 20% | 19% | 11% |
|  | Regress from small (6-9 mm) to diminutive (<6 mm) size | 48% | 78% | 34% |
|  | Regress from large (>=10 mm) to small (6-9 mm) size | 17% | 43% | 18% |
| *Probability of developing high-grade dysplasia by adenoma size ^a^* | | | | |
|  | Diminutive adenoma | 0.4% | 0.9% | 0.4% |
|  | Small adenoma | 0.7% | 0.9% | 0.4% |
|  | Large adenoma | 0.8% | 1.2% | 0.5% |
| *Probability of developed villous component ^a^* | | | |  |
|  | Diminutive adenoma | 0.4% | 0.7% | 0.4% |
|  | Small adenoma | 3.5% | 3.3% | 1.3% |
|  | Large adenoma | 5.4% | 8.7% | 5.7% |
| *Probability of polyp with sessile shape by section of bowel ^b^* | | | | |
|  | Cecum | 87.3% | As baseline | As baseline |
|  | Ascending colon | 86.5% | As baseline | As baseline |
|  | Transverse colon | 86.2% | As baseline | As baseline |
|  | Descending colon | 85.7% | As baseline | As baseline |
|  | Sigmoid | 66.5% | As baseline | As baseline |
|  | Rectum | 66.2% | As baseline | As baseline |
| *Probability of adenoma with pedunculated shape by section of bowel ^b^* | | | | |
|  | Cecum | 1.3% | As baseline | As baseline |
|  | Ascending colon | 8.2% | As baseline | As baseline |
|  | Transverse colon | 5.2% | As baseline | As baseline |
|  | Descending colon | 13.1% | As baseline | As baseline |
|  | Sigmoid | 30.3% | As baseline | As baseline |
|  | Rectum | 32.3% | As baseline | As baseline |
| *Probability of adenoma with flat shape by section of bowel ^b^* | | | |  |
|  | Cecum | 11.4% | As baseline | As baseline |
|  | Ascending colon | 5.3% | As baseline | As baseline |
|  | Transverse colon | 8.6% | As baseline | As baseline |
|  | Descending colon | 1.2% | As baseline | As baseline |
|  | Sigmoid | 3.2% | As baseline | As baseline |
|  | Rectum | 1.5% | As baseline | As baseline |
| *Distribution of adenoma by section of bowel, 20-64 years ^b^* | | | | |
|  | Cecum | 10.9% | As baseline | As baseline |
|  | Ascending colon | 22.1% | As baseline | As baseline |
|  | Transverse colon | 13.7% | As baseline | As baseline |
|  | Descending colon | 10.9% | As baseline | As baseline |
|  | Sigmoid | 22.9% | As baseline | As baseline |
|  | Rectum | 19.5% | As baseline | As baseline |
| *Distribution of adenoma by section of bowel, 65+ years ^b^* | | | | |
|  | Cecum | 11.1% | As baseline | As baseline |
|  | Ascending colon | 26.3% | As baseline | As baseline |
|  | Transverse colon | 19.8% | As baseline | As baseline |
|  | Descending colon | 11.5% | As baseline | As baseline |
|  | Sigmoid | 15.7% | As baseline | As baseline |
|  | Rectum | 15.6% | As baseline | As baseline |
| *Probability of advanced adenoma progress into stage 1 non-symptomatic cancer ^a^* | | | | |
|  | Male, colon cancer | 2.1% | As baseline | As baseline |
|  | Male, rectum cancer | 5.1% | As baseline | As baseline |
|  | Female, colon cancer | 2.0% | As baseline | As baseline |
|  | Female, rectum cancer | 3.5% | As baseline | As baseline |
| ***Serrated pathway*** | | | | |
| *Hyperplastic polyp incidence rate ^a^* | | | | |
|  | Male, 20-39 years | 0.05% | 0.10% | 0.03% |
|  | Male, 40-49 years | 0.15% | 0.21% | 0.05% |
|  | Male, 50-54 years | 0.13% | 0.16% | 0.07% |
|  | Male, 55-59 years | 0.24% | 0.28% | 0.13% |
|  | Male, 60-64 years | 0.24% | 0.28% | 0.13% |
|  | Male, 65-69 years | 0.24% | 0.28% | 0.13% |
|  | Male, 70-74 years | 0.24% | 0.28% | 0.13% |
|  | Male, 75+ years | 0.24% | 0.28% | 0.13% |
|  | Female, 20-39 years | 0.03% | 0.07% | 0.02% |
|  | Female, 40-49 years | 0.09% | 0.15% | 0.04% |
|  | Female, 50-54 years | 0.08% | 0.11% | 0.05% |
|  | Female, 55-59 years | 0.14% | 0.20% | 0.09% |
|  | Female, 60-64 years | 0.14% | 0.20% | 0.09% |
|  | Female, 65-69 years | 0.14% | 0.20% | 0.09% |
|  | Female, 70-74 years | 0.51% | 0.67% | 0.27% |
|  | Female, 75+ years | 0.51% | 0.67% | 0.27% |
| *Sessile serrated polyp incidence rate ^a^* | | | | |
|  | Male, 20-39 years | 0.092% | 0.169% | 0.066% |
|  | Male, 40-49 years | 0.032% | 0.117% | 0.030% |
|  | Male, 50-54 years | 0.009% | 0.081% | 0.015% |
|  | Male, 55-59 years | 0.009% | 0.049% | 0.008% |
|  | Male, 60-64 years | 0.009% | 0.049% | 0.008% |
|  | Male, 65-69 years | 0.009% | 0.049% | 0.008% |
|  | Male, 70-74 years | 0.009% | 0.049% | 0.008% |
|  | Male, 75+ years | 0.009% | 0.049% | 0.008% |
|  | Female, 20-39 years | 0.067% | 0.117% | 0.045% |
|  | Female, 40-49 years | 0.023% | 0.079% | 0.022% |
|  | Female, 50-54 years | 0.007% | 0.056% | 0.011% |
|  | Female, 55-59 years | 0.006% | 0.035% | 0.006% |
|  | Female, 60-64 years | 0.006% | 0.035% | 0.006% |
|  | Female, 65-69 years | 0.007% | 0.036% | 0.006% |
|  | Female, 70-74 years | 0.007% | 0.036% | 0.006% |
|  | Female, 75+ years | 0.007% | 0.036% | 0.006% |
| *Hyperplastic polyps size progression and regression rate ^a^* | | | | |
|  | Progress from small (<10 mm) to large (>=10 mm) size | 2% | 3% | 1% |
|  | Regress from small (<10 mm) size to none | 7% | 11% | 3% |
|  | Regress from large (>= 10 mm) to small (<10 mm) size | 29% | 44% | 22% |
| *Sessile serrate polyp size progression and regression rate ^a^* | | | | |
|  | Progress from small (<10 mm) to large (>=10 mm) size | 3% | 3% | 2% |
|  | Regress from large (>= 10mm) to small (<10 mm) size | 33% | 33% | 17% |
| *Distribution of hyperplastics polyps by section of bowel ^b^* | | | | |
|  | Cecum | 4% | As baseline | As baseline |
|  | Ascending colon | 12% | As baseline | As baseline |
|  | Transverse colon | 9% | As baseline | As baseline |
|  | Descending colon | 7% | As baseline | As baseline |
|  | Sigmoid | 26% | As baseline | As baseline |
|  | Rectum | 42% | As baseline | As baseline |
| *Distribution of sessile serrated polyp by section of bowel ^b^* | | | | |
|  | Cecum | 12% | As baseline | As baseline |
|  | Ascending colon | 28% | As baseline | As baseline |
|  | Transverse colon | 20% | As baseline | As baseline |
|  | Descending colon | 7% | As baseline | As baseline |
|  | Sigmoid | 13% | As baseline | As baseline |
|  | Rectum | 21% | As baseline | As baseline |
| *Probability of sessile serrated polyp (any size) progress into stage 1 non-symptomatic cancer ^a^* | | | | |
|  | Male, colon cancer | 0.17% | As baseline | As baseline |
|  | Male, rectum cancer | 0.43% | As baseline | As baseline |
|  | Female, colon cancer | 0.16% | As baseline | As baseline |
|  | Female, rectum cancer | 0.29% | As baseline | As baseline |

^a^ Annual rate

^b^ Input parameters not outcome of calibration

Figure B1 - Modelled age-specific colorectal cancer incidence and mortality rates associated with the base case, the least aggressive precancer natural history assumptions, and the most aggressive precancer natural history assumptions when screening was no available

| 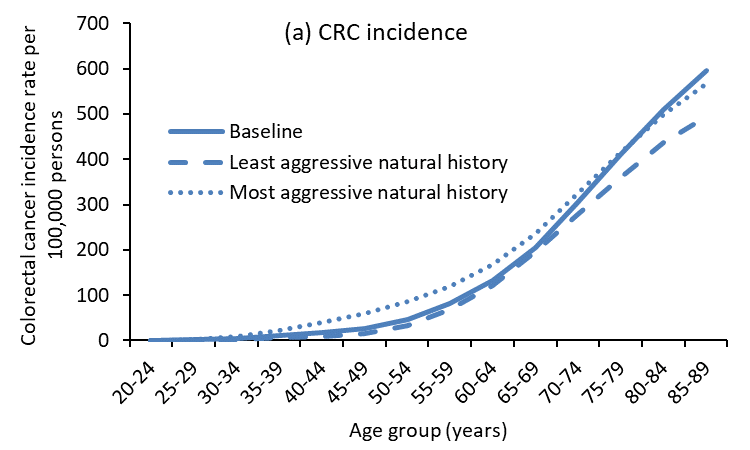 | 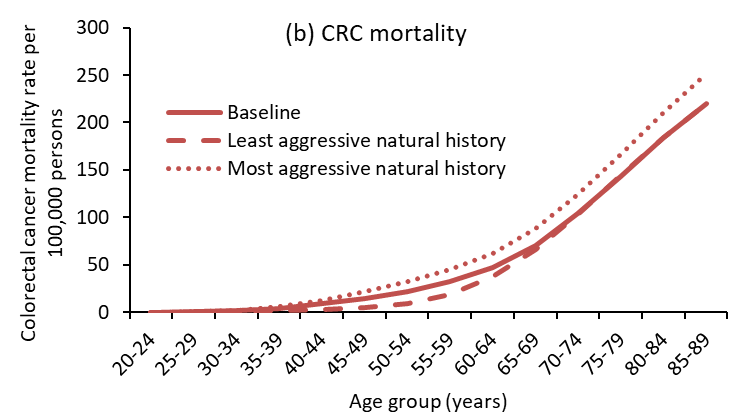 |
| --- | --- |

## Immunochemical Faecal Occult Blood Test (iFOBT) characteristics

An iFOBT is used as the primary screening test by NBCSP in Australia.[3] The NBCSP used *Magstream HemSp* with a rabbit serum buffer, manufactured by Fujirebio Inc (Tokyo), as the screening test in 2006-2017.[4] The Program followed the manufacturer's recommendations to use 20 ng Hb/ml buffer (equivalent to 20 ug Hb/g faeces) as the cut-off for test positivity and to collect two faecal samples from participants. In January 2018, the Program switched-over to a new iFOBT kit (an Eiken kit – OC Sensor Auto-sampling Bottle 3 – using the Pledia analyser). However, the modelled test characteristics of iFOBT in this study was derived from NBCSP data in 2006-2014, when Magstream HemSp was used as the single program-wide iFOBT technology.

The model assumed 1.6% of all iFOBT tests returned were incorrectly completed based on observed data in 2013-14.[5] The modelled test characteristics of a correctly completed iFOBT were obtained via calibrating to (i) iFOBT positive rates (the proportion of participants with a positive result out of all participants who returned a valid FOBT kit) observed among the men and women invited to participate in NBCSP, and (ii) the colonoscopy outcomes among those with a positive iFOBT result in the period between 2006 and 2014. The relevant model calibration outcomes were reported elsewhere.[2] Table B3 shows the modelled lesion-specific iFOBT test positive rate for baseline.

Table B3 - Modelled lesion-specific test positive rate of iFOBT

| **Category** | **Modelled iFOBT positive rate** |
| --- | --- |
| Background rate in all individuals (per individual) ^a^ | 5.0% |
| Additional positive rate per adenoma 1-5 mm ^b^ | 0.7% |
| Additional positive rate per adenoma 6-9 mm ^b^ | 11.0% |
| Additional positive rate per adenoma >10 mm ^b^ | 35.0% |
| Additional positive rate per HP | 0% |
| Additional positive rate per SSP | 0% |
| Additional positive rate for stage 1 CRC | 40.0% |
| Additional positive rate for stage 2 CRC | 65.0% |
| Additional positive rate for stage 3 CRC | 75.0% |
| Additional positive rate for stage 4 CRC | 75.0% |

*CRC- colorectal cancer; HP- hyperplastic polyp; SSP – sessile serrate polyp*

*^a^ A background positive rate was assumed for all individuals (including perfectly healthy individuals who have no polyps or cancer)*

*^b^ Same positive rate was assumed for adenoma within the same size category regardless of the histopathology characteristic of the adenoma (i.e. with or without high-grade dysplasia, and with/without villous architecture)*

Table B4 provides a summary of the modelled per-person iFOBT test sensitivity and specificity that are associated with the modelled lesion-specific test positive rate of iFOBT summarised in Table B3. These per-person iFOBT test sensitivity and specificity summarised in Table B3 were estimated from iFOBT and colonoscopy outcomes of a group of never-screened individuals aged 50-74 years who underwent one round of screening using both iFOBT (as per test characteristics provided in Table B3) and colonoscopy in the model. The following definitions were used for the test sensitivity and test specificity calculations:

- Individuals who had a positive iFOBT result were counted as true positive if their colonoscopy findings met the criteria defined for each category of colonoscopy outcome specified in Table B4, i.e. any adenoma, adenoma >5mm, >10mm, respectively; otherwise, the individuals were counted as false positive.
- Individuals who had a negative iFOBT result were counted as false negative if their colonoscopy outcome met the criteria defined for each category of colonoscopy outcome specified in Table B4; otherwise, the individuals were counted as true negative.
- Test sensitivity was calculated by dividing true positive by the sum of true positive and false negative.
- Test specificity was calculated by dividing the true negative by the sum of true negative and false positive.

Table B4 - Modelled test sensitivity and specificity of iFOBT (per person)

| Colonoscopy outcome | Baseline | |
| --- | --- | --- |
|  | Sens | Spec |
| Any adenoma | 15.2% | 94.8% |
| Adenoma > 5mm | 30.2% | 94.6% |
| Adenoma >=10mm | 41.5% | 94.1% |
| CRC | 58.6% | - |

CRC- colorectal cancer; Sens- sensitivity; Spec-specificity

In a publication by the Australian Institute of Health and Welfare (AIHW), the sensitivity of iFOBT was reported to be 83% for colorectal cancer (any stage) detection within the NBCSP.[6] This ‘program sensitivity’ was measured based on screen-detected cancers individuals with positive iFOBT result (as true positive outcome of the iFOBT) and the subsequent interval cancers diagnosed within 2 years in people with a negative iFOBT at screening (as the false negative of iFOBT). Using this definition, the test sensitivity for colorectal cancer detection of the modelled base case iFOBT was estimated to be 82.5%, consistent with the AIHW findings.

## Colonoscopy test characteristics

The modelled lesion-specific positive rates of colonoscopy are summarised in Table B5. The modelled positive rate of colonoscopy for conventional adenomas, hyperplastic polyps and sessile serrated polyps were based on the findings of a 2006 systematic review by van Rijn and colleagues.[7] The study found the colonoscopy miss rate for large adenoma (>= 10mm) was 2.1% (95% confidence interval [CI]: 0.3-7.3%), for small adenoma (5 -10mm) was 13% (95% CI: 8.0-18.0%), and for diminutive adenomas (< 5 mm) was 26% (95% CI: 27-35%). The modelled detection rate of colorectal cancer (any stage) was based on the findings of a 2011 systematic review and meta-analysis conducted by Pickhardt and colleagues, which reported a sensitivity of 94.7% (95% CI: 90.4-97.2%) for colorectal cancer detection.[8] The model assumed the end of caecum was reached in all colonoscopy procedures and the test specificity for detecting polyps and colorectal cancer of colonoscopy was 100%. That means individuals who have no polyp or cancer in the bowel would always have a negative colonoscopy outcome in the model. Polypectomy was assumed to be performed on all polyps detected by colonoscopy, except hyperplastic polyps, with 100% success rate. Based on the NBCSP data observed in 2013-2014, 0.27% individuals undergoing colonoscopy in the model were assumed to experience colonoscopy-related-non-fatal adverse events.[5]

Table B5 - Modelled lesion-specific positive rate of colonoscopy

| Polyp/cancer | Base case |
| --- | --- |
|  |  |
| Diminutive adenoma (<5mm) | 79.0% |
| Small adenoma (6-9mm) | 85.0% |
| Large adenoma (>= 10mm) | 92.0% |
| Sessile serrated polyp (any size) | 78.0% |
| Hyperplastic polyp (<10 mm) | 78.0% |
| Hyperplastic polyp (>=10 mm) | 92.0% |
| Colorectal cancer (any stage) | 95.0% |

Taking both the test characteristics of iFOBT and colonoscopy into account, the modelled colonoscopy outcomes among individuals with positive iFOBTs were validated to data observed in the NBCSP in the period between 2006 and 2014.[5, 9-13] The model validation outcomes were reported elsewhere.[2] Two alternative colonoscopy test characteristics assuming colonoscopy detection rates increased/decreased by 10% were assessed in sensitivity analysis.

# Appendix C – Supplementary analysis on the addition of screening colonoscopies to the NBCSP

An extreme scenario designed to evaluate the potential maximum number of CRC deaths which could be prevented via population-level screening was modelled. This scenario assumed supplementary screening colonoscopies were offered to people aged 40 and 60 years in parallel to the current NBCSP. Colonoscopy is considered a gold-standard in bowel screening efficacy, with a recent study estimating that once-off colonoscopy screening alongside iFOBT screening could reduce CRC mortality by 78%, although it would not be cost-effective.[14]

For the supplementary analysis iFOBT screening was assumed to have a 90% participation rate from 2020, and colonoscopy follow-up rates were modelled at 90%, as in Scenario 3 of the main analysis. Compliance to the screening colonoscopies was modelled at 90%.

This scenario was estimated to reduce CRC incidence and mortality ASRs to 21.2 and 4.1 per 100,000 respectively by 2040. The addition of colonoscopies would be associated with a significant increase in the overall cost (40.1% increase vs no screening annually) and number of colonoscopies (615% increase over the period of 2020-2040) versus the comparator, the NBCSP with currently observed participation rates. It would also be associated with a very high NNC of 348.5 per CRC death prevented (about 3.5 times higher than Scenarios 1-3). This scenario was not found to be cost-effective. The full results are shown in Table C3. Note that the MTI was not calculated for Scenario 4 because it was not cost-effective.

**Table C3** – Outcomes for supplementary analysis: screening colonoscopies at age 40 and 60 alongside NBCSP iFOBT screening. iFOBT screening participation, colonoscopy follow-up rates, and participation in screening colonoscopies were all assumed to be 90%. All costs throughout are in AUD.

| **Colorectal Cancer Cases** | **Total 2020-2040** | 294,800 |
| --- | --- | --- |
|  | **vs Comparator (% change)** | -121,600 (-29.2%) |
| **Colorectal Cancer Deaths** | **Total 2020-2040** | 79,100 |
|  | **vs Comparator (% change)** | -52,500 (-39.9%) |
| **Number of colonoscopies^a^** | **Total 2020-2040** | 21,258,800 |
|  | **vs Comparator (% change)** | 18,288,200 (615.6%) |
| **Colonoscopy-related adverse events** | **Total 2020-2040** | 57,400 |
|  | **vs Comparator (% change)** | 49,400 (615.9%) |
| **Total costs^b^** | **Total 2020-2040** | $64.52 |
|  | **vs Comparator (% change)** | $23.73 (58.2%) |
| **Number needed to colonoscope per CRC death prevented** | | 348.5 |
| **Undiscounted life-years per person** | | 61.006 |
| **Undiscounted lifetime cost per person** | | $8,634 |
| **Discounted life-years per person^c^** | | 37.444 |
| **Discounted lifetime cost per person^c^** | | $3,872 |
| **CER vs no change to screening participation** | | $64,753/LYS |

*^a^* *Includes both colonoscopy assessments performed to follow-up individuals with positive iFOBT results, and colonoscopies to provide surveillance for individuals with the removal of at least one adenoma and/or sessile serrated polyp. Out-of-program colonoscopies were not included in the model estimates.*

*^b^* *Costs considered are the undiscounted costs associated with sending the iFOBT kits, laboratory analysis of the completed iFOBT samples, general practitioner visit for follow-up of positive iFOBT results, colonoscopy procedures with/without adverse events (and polypectomy if required) to follow-up positive iFOBT result and to provide surveillance, and colorectal cancer treatments.*

*^c^Discounted life-years per person and discounted costs are calculated with a 5% discount rate per year. The cost-effectiveness ratio (CER) is then calculated as the additional cost divided by the additional life-years vs the comparator.*

# Appendix D – Sensitivity analysis with more and less aggressive precancer natural history

For sensitivity analysis, the cost-effectiveness ratios were recalculated using simulations that assumed a more aggressive and less aggressive natural history pathway (see Table B2). These parameter sets reflect uncertainties inherent in the *Policy1-Bowel* model. The CERs are still cost-effective or cost-saving under these alternative assumptions, and are shown in Table D1 and Table D3. The investment thresholds analogous to Table 4 in the full paper are shown in Table D2 and Table D4.

Table D1 – More aggressive single cohort outcomes for 1980 cohort with full biennial screening. The discounted life-years per person and discounted costs are calculated with a 5% discount rate per year. The cost-effectiveness ratio (CER) is then calculated as the additional cost divided by the additional life-years vs the comparator. Scenario 1 is cost-saving and more effective. The maximum additional discounted cost is shown; this is the maximum discounted cost per person for each scenario while remaining below a willingness-to-pay threshold of $10,000/LYS, $20,000/LYS, and $30,000/LYS.

|  | **Discounted life-years per person** | **Discounted lifetime cost per person** | **Interim CER** *^a^* |
| --- | --- | --- | --- |
|  |  |  |  |
| **Comparator** | 37.3821 | $2,302 | - |
| **Scenario 1** | 37.3849 | $2,278 | Cost-saving^b^ |
| **Scenario 2** | 37.4015 | $2,195 | Cost-saving^b^ |
| **Scenario 3** | 37.4061 | $2,208 | Cost-saving^b^ |

*^a^ CER without considering costs related to health promotion interventions.*

*^b^ All scenarios are cost-saving and more effective than the comparator*.

Table D2 – The maximum additional cost for each scenario while remaining under the reference WTP thresholds with more aggressive precancer natural history assumptions. The maximum investment per person is the additional discounted lifetime costs; this can be thought of as a one-off undiscounted cost age 40, or a discounted cost spread across a lifetime. The maximum total is estimated using the Australian 2020 population, and assuming costs occur at age 50. These results are presented for willingness-to-pay thresholds of $10,000/LYS, $20,000/LYS, and $30,000/LYS.

|  | **Maximum investment per person under WTP threshold (AUD)** | | | **Maximum investment total under WTP threshold (AUD, millions)** | | |
| --- | --- | --- | --- | --- | --- | --- |
|  | **$10,000/LYS** | **$20,000/LYS** | **$30,000/LYS** | **$10,000/LYS** | **$20,000/LYS** | **$30,000/LYS** |
| **Scenario 1** | $53.49 | $82.21 | $110.93 | $18.59 | $28.58 | $38.56 |
| **Scenario 2** | $301.97 | $496.23 | $690.49 | $104.97 | $172.49 | $240.01 |
| **Scenario 3** | $334.11 | $574.05 | $813.99 | $116.14 | $199.54 | $282.94 |

Table D3 – Less aggressive natural history single cohort outcomes for 1980 cohort with full biennial screening. The discounted life-years per person and discounted costs are calculated with a 5% discount rate per year. The cost-effectiveness ratio (CER) is then calculated as the additional cost divided by the additional life-years vs the comparator. Scenario 1 is cost-saving and more effective. The maximum additional discounted cost is shown; this is the maximum discounted cost per person for each scenario while remaining below a willingness-to-pay threshold of $10,000/LYS, $20,000/LYS, and $30,000/LYS.

|  | **Discounted life-years per person** | **Discounted lifetime cost per person** | **Interim CER** *^a^* |
| --- | --- | --- | --- |
|  |  |  |  |
| **Comparator** | 37.4253 | $1,561 | - |
| **Scenario 1** | 37.4273 | $1,548 | Cost-saving^b^ |
| **Scenario 2** | 37.4374 | $1,533 | $317/LYS |
| **Scenario 3** | 37.4409 | $1,562 | $93/LYS |

*^a^ CER without considering costs related to health promotion interventions.*

*^b^ Scenario 1 is cost-saving and more effective than the comparator*.

Table D4 – The maximum additional cost for each scenario while remaining under the reference WTP thresholds with less aggressive precancer natural history assumptions. The maximum investment per person is the additional discounted lifetime costs; this can be thought of as a one-off undiscounted cost age 40, or a discounted cost spread across a lifetime. The maximum total is estimated using the Australian 2020 population, and assuming costs occur at age 40. These results are presented for willingness-to-pay thresholds of $10,000/LYS, $20,000/LYS, and $30,000/LYS.

|  | **Maximum investment per person under WTP threshold (AUD)** | | | **Maximum investment total under WTP threshold (AUD, millions)** | | |
| --- | --- | --- | --- | --- | --- | --- |
|  | **$10,000/LYS** | **$20,000/LYS** | **$30,000/LYS** | **$10,000/LYS** | **$20,000/LYS** | **$30,000/LYS** |
| **Scenario 1** | $32.76 | $52.30 | $71.83 | $11.39 | $18.18 | $24.97 |
| **Scenario 2** | $149.25 | $270.33 | $391.41 | $51.88 | $93.97 | $136.05 |
| **Scenario 3** | $154.39 | $310.24 | $466.08 | $53.67 | $107.84 | $162.01 |

1. Stoop, E.M., et al., *Participation and yield of colonoscopy versus non-cathartic CT colonography in population-based screening for colorectal cancer: a randomised controlled trial.* Lancet Oncol, 2012. **13**(1): p. 55-64.

2. Lew, J.B., et al., *Long-term evaluation of benefits, harms, and cost-effectiveness of the National Bowel Cancer Screening Program in Australia: a modelling study.* Lancet Public Health, 2017. **2**: p. e331-e340.

3. Australian Government Department of Health, *National Bowel Cancer Screening Program - About the Program.* 2014. **2014**(20 May).

4. Binefa, G., et al., *Colorectal cancer screening programme in Spain: results of key performance indicators after five rounds (2000–2012).* Scientific reports, 2016. **6**: p. 19532.

5. Australian Institute of Health and Welfare, *National Bowel Cancer Screening Program: monitoring report 2013-14*. 2015: Cancer series No. 94. Cat. no. CAN 94.

6. Australian Institute of Health and Welfare and H. Australian Government Department of, *Analysis of colorectal cancer outcomes for the Australian National Bowel Cancer Screening Program.* Asia Pac J Clin Oncol, 2016. **12**(1): p. 22-32.

7. van Rijn, J.C., et al., *Polyp miss rate determined by tandem colonoscopy: a systematic review.* Am J Gastroenterol, 2006. **101**(2): p. 343-350.

8. Pickhardt, P.J., et al., *Colorectal cancer: CT colonography and colonoscopy for detection--systematic review and meta-analysis.* Radiology, 2011. **259**(2): p. 393-405.

9. Australian Institute of Health and Welfare, *National Bowel Cancer Screening Program monitoring report: 2012-2013. Cancer series No. 84. Cat. no. CAN 81*. 2014, AIHW: Canberra.

10. Australian Institute of Health and Welfare and Australian Institute of Health and Ageing, *National Bowel Cancer Screening Program: monitoring report 2008. Cancer series no. 44. Cat. no. CAN 40*. 2008, AIHW: Canberra.

11. Australian Institute of Health and Welfare, *National Bowel Cancer Screening Program: annual monitoring report 2009. Cancer series no. 49. Cat. no. CAN 45*. 2009, Australian Institute of Health and Welfare; Australian Gevernment Department of Health and Ageing; National Bowel Cancer Screening Program: Canberra.

12. Australian Institute of Health and Welfare, *National Bowel Cancer Screening Program monitoring report: phase 2, July 2008-June 2011. Cancer series no. 65. Cat no. CAN 61*. 2012, AIHW: Canberra.

13. Australian Institute of Health and Welfare, *National Bowel Cancer Screening Program monitoring report: July 2011-June 2012. Cancer series no. 75. Cat. no. CAN 71*. 2013, AIHW: Canberra.

14. Lew, J.B., et al., *Evaluation of the benefits, harms and cost-effectiveness of potential alternatives to iFOBT testing for colorectal cancer screening in Australia.* Int J Cancer, 2018. **143**(2): p. 269-282.
